# Diagnostic Values of Electrochemiluminescent Detection of Urinary CYFRA21‐1 and FDP and Their Combined Detection in Bladder Cancer

**DOI:** 10.1002/cam4.71056

**Published:** 2025-08-01

**Authors:** Zhuoran Li, Lu Xia, Yamin Li, Tao Huang, Ning Yang, Shannai Li, Yinyin Luo, Yaoyao Yang, Jian Zhang, Song Wu, Qifang Lei

**Affiliations:** ^1^ Department of Urology, Medical School, South China Hospital Shenzhen University Shenzhen PR China; ^2^ Shenzhen Luohu People's Hospital Shenzhen PR China; ^3^ Department of Central Laboratory Binzhou People's Hospital Binzhou PR China; ^4^ Shenzhen Lifotronic Technology Co., Ltd. Shenzhen PR China

**Keywords:** bladder cancer, combined detection, CYFRA21‐1, electrochemiluminescence, FDP

## Abstract

**Background:**

Bladder cancer is the most common malignant tumor in the urinary system. The acquisition of urine samples has the advantages of being rapid and painless, and it can directly contact the lesions. It is an ideal source of non‐invasive tumor markers. The problems with urine markers are low specificity, the propensity for false positives or missed detections, and there is no unified diagnostic standard to measure the test results, which limits the clinical application of urine markers. In this study, we evaluate the diagnostic values of Cytokeratin 19 fragment (CYFRA21‐1), Fibrin/fibrinogen degradation products (FDP) and CYFRA21‐1 + FDP in bladder cancer, with the objective to expand the application scope of urinary tumor markers in the diagnosis of bladder cancer.

**Methods:**

We evaluated the performances of the CYFRA21‐1 assay kit (electrochemiluminescence) and/or FDP assay kit (electrochemiluminescence) in detecting bladder cancer. CYFRA21‐1 and FDP levels were determined in urine samples from 467 participants in South China Hospital of Shenzhen University and Shenzhen Luohu People's Hospital. We performed performance validation of the CYFRA21‐1 and FDP kits, established normal biological reference intervals, and finally analyzed the data to assess their diagnostic efficacies alone or in combination.

**Results:**

The bladder cancer group had significantly higher urinary levels of CYFRA21‐1 and FDP than the control group. The areas under the receiver operating characteristic curves (ROC‐AUCs) of CYFRA21‐1, FDP, and CYFRA21‐1 + FDP were 0.929, 0.857, and 0.935, respectively, for discriminating bladder cancer cases from controls. The ROC‐AUC of CYFRA21‐1 and FDP in combination was higher than that of any index alone.

**Conclusions:**

Both CYFRA21‐1 and FDP have good diagnostic values in predicting bladder cancer. In addition, combined detection of urinary CYFRA21‐1 and FDP by electrochemiluminescence has better differentiation ability compared with each index alone and may be used for early screening and recurrence monitoring in bladder cancer.

## 1 Introduction

In 2020, there were 570,000 new cases of bladder cancer and 210,000 related deaths around the world [1]. In 2022, the number of new bladder cancer cases in China reached 92,900 [2]. Early‐stage bladder cancer is characterized by insidiousness. According to the degree of bladder cancer invasion, this malignancy can be divided into superficial and invasive types, with invasive bladder cancer being highly malignant and difficult to treat, with a recurrence rate as high as 80% [3, 4]. Timely diagnosis and treatment can increase the 5‐year survival rate of patients to over 90% [4]. In current clinical practice, cystoscopy and urine exfoliative cytology remain the main detection methods in bladder cancer [5]. It is worth noting that the sensitivities of cystoscopy and urine exfoliative cytology depend on the pathological stage of bladder cancer, and both techniques have low sensitivities for low‐grade bladder cancer [6]. It is urgent to explore urine bladder tumor markers with high sensitivity and specificity, which can be used in early screening, recurrence monitoring, and curative effect assessment in bladder cancer. Urine sampling has the advantages of speediness and painlessness. It is an ideal source of non‐invasive tumor markers and an ideal index for auxiliary diagnosis of bladder cancer.

Keratin 19 normally exists in serum in the form of an oligomer in very low amounts; in bladder cancer, CK19 amounts increase, cancer cells undergo necrosis and exfoliation, and CK19 is released in the form of dissolved fragments [7]. Cytokeratin 19 fragment (CYFRA21‐1) is a soluble fragment of cytokeratin 19 used to observe the curative effect and monitor tumor recurrence in non‐small cell lung cancer [8, 9]. In a previous study, we evaluated the performance of the CYFRA21‐1 kit and its clinical diagnostic value in our first preliminary experiment [10].

Fibrin/fibrinogen degradation products (FDP) is a urine marker approved by the Food and Drug Administration (FDA) for clinical use [11]. FDP has certain clinical significance in the diagnosis of primary hyperfibrinolysis [12, 13, 14]. Coagulation factors released by bladder tumor cells convert fibrinogen into fibrin deposits, which are then degraded into FDP [12, 15, 16, 17]. Classical FDP detection methods include latex agglutination and enzyme‐linked immunosorbent assay (ELISA). Electrochemiluminescence (ECLIA) detection of FDP has the advantages of simple operation and short assay time, which reduces the manual operation error [18]. Previous studies have demonstrated abnormal urine FDP amounts in bladder cancer [15, 16, 17], but no study has applied ECLIA for FDP detection [19].

This study aimed to evaluate the performance of CYFRA21‐1 and FDP kit, and assess the diagnostic performances of ECLIA detections of FDP and/or CYFRA21‐1, to identify a simple, rapid, and reliable method for the diagnosis of bladder cancer.

## 2 Materials and Methods

### 2.1 Study Design and Population

**FIGURE 1:**
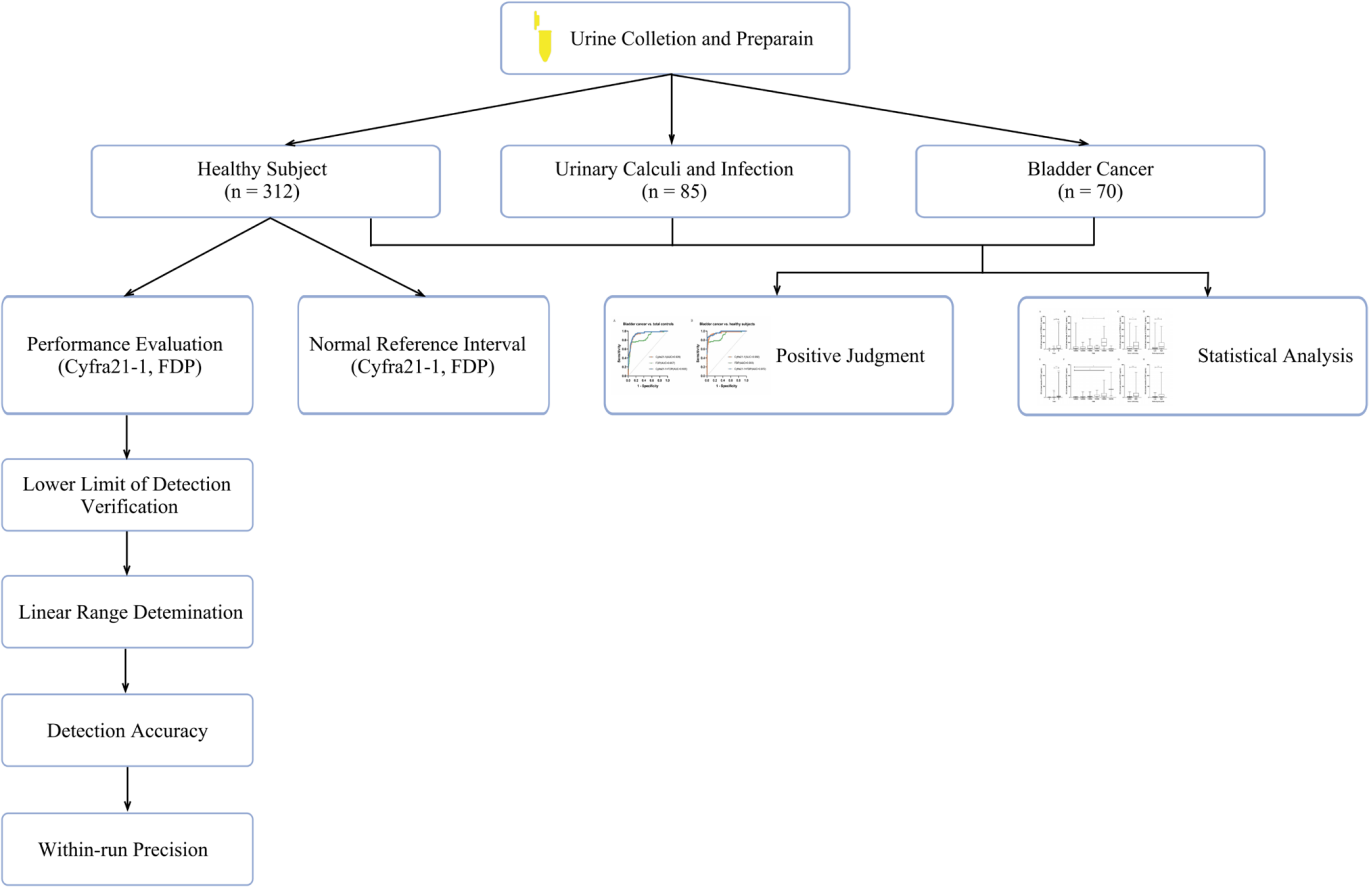
The workflow of diagnostic values of electrochemiluminescent detection of urinary CYFRA21‐1 and FDP and their combined detection in bladder cancer. Phase I: Analytical validation (*n* = 312 healthy subjects) assessing LLOD verification, linear range determination, accuracy evaluation, and within‐run precision evaluation. Phase II: Determined the critical value (*n* = 312 healthy subjects). Phase III: Positive judgment in 467 urine samples (control/blander cancer = 397/70). Phase IV: Statistical analysis (Ta/Tis/T1/T2/T3/T4 = 14/18/16/8/11/3, NMIBC/MIBC = 48/22, pathological low/pathological high = 32/38).

A total of 467 urine samples were collected between July 2020 and May 2022 from patients who visited Shenzhen Luohu People's Hospital for medical treatment, without malignant diseases other than bladder cancer. Voided mid‐stream random urine samples were collected, and CYFRA21‐1 and FDP amounts were measured. The subjects were divided into three groups, including bladder cancer cases (*n* = 70), healthy subjects (*n* = 312), urinary calculi and infection cases (*n* = 85). In this study, the performance of the kit was evaluated, including lower limit of detection (LLOD) verification, linear range determination, accuracy evaluation and within‐run precision evaluation. We determined the critical value to distinguish bladder cancer according to the ROC curve. All bladder cancer patients were diagnosed based on histological examination and staged as superficial (pTa or pT1) or muscle invasive (pT2, pT3 or pT4) cancer according to TNM criteria [20]. Histological grade was assessed following the WHO tumor grading system (2017 AJCC 8th edition) [21]. We statistically analyzed the correlation between CYFRA21‐1 and FDP and their combined detection and TNM stage, tumor infiltrating cells and pathological grade of bladder cancer. The specific experimental flow is shown in Figure 1. The study was approved by the Shenzhen Luohu People's Hospital's ethics committee (2019‐LHQRMYY‐LL‐053).

#### 2.1.1 Inclusion Criteria

- Aormal blood routine, urine routine, and liver and kidney blood lipid indexes.
- Diagnosis of bladder urothelial carcinoma, urinary calculi, or urinary tract infection.

#### 2.1.2 Exclusion Criteria

- Incomplete medical records.
- Menstruation, pregnancy or lactation in women.
- Microbial contamination of samples.

### 2.2 Reagents and Instruments

An Automatic chemiluminescence meter eCL8000 (eCL8000, Shenzhen Lifotronic Technology Co. Ltd.; China) was utilized for detection. Cytokeratin 19 fragment (CYFRA21‐1) assay kit (electrochemiluminescence; ZM0922001) and Fibrin/fibrinogen degradation products (FDP) assay kit (electrochemiluminescence; 20,220,518,001) were from Shenzhen Lifotronic Technology Co. Ltd. FDP antigen was from Changzhou ai fu kang biological technology Co. Ltd. (China, GK‐052).

### 2.3 CYFRA21‐1 Performance Evaluation

#### 2.3.1 CYFRA21‐1's LLOD Verification

A total of 5 low—value samples (A1–A5) with concentrations near the detection limit were measured, with each sample being measured 5 times. The measurement results were sorted by size and must meet the following requirements:

- Number of detection results below 0.1 ng/mL ≤ 3;
- No measurement results higher than 3.3 ng/mL.

#### 2.3.2 CYFRA21‐1 Linear Range Determination

A high concentration antigen solution (500 ng/mL, used as a high value sample) and a low concentration (0.1 ng/mL) antigen solution were proportionally diluted to generate six concentration samples (X1–X6), and the test was repeated twice for each concentration. The results were averaged, and a theoretical value for each sample was calculated according to the dilution ratio (the upper limit of linear range samples would be the mean of the results of three repeated determinations considered the theoretical value). A correlation coefficient, r, was obtained by regression analysis between measured and theoretical values.

Acceptance criteria were: kit's detection interval between 0.1 and 500 ng/mL; *r* ≥ 0.990.

#### 2.3.3 Measurement of CYFRA21‐1 Detection Accuracy

A known sample A at high concentration was added to sample B at low concentration (the concentration of B should be no higher than 1/10–1/8 of the concentration of A, depending on the specificity of the A concentration), and the volume ratio between samples A and B was 1:9 or less. Both samples were mixed to generate samples with the correct ratio at high and low concentrations (Z1, Z2). The relative deviations were calculated by averaging the data of two replicates in three fully automated chemiluminescence assays with different lot numbers (instrument no. i05a20601238 for instrument No. 1, instrument no. i05a20601229 for instrument No. 2, and instrument no. i05a17900146 for instrument No. 3). The acceptance criterion was a relative deviation not above ±10%.

#### 2.3.4 Measurement of CYFRA21‐1 Within‐Run Precision

Precision high‐value and low‐value specimens were tested with reagents and calibrators of the same lot numbers, and the coefficient of variation (CV) was calculated by testing 10 consecutive times. The acceptance criterion was CV ≤ 6.0%.

### 2.4 FDP Performance Testing

#### 2.4.1 FDP's LLOD Verification

Five low value samples (A6–A10) whose concentrations were close to the detection limit were measured 5 times each. The measurement results were sorted according to size, and the results should meet the following requirements:

- Number of test results less than 1.0 μg/mL ≤ 3.
- No measurement above 2.5 μg/mL.

#### 2.4.2 FDP Linear Range Assessment

A high concentration antigen solution (80 μg/mL, high value sample) and a low concentration antigen solution (1.0 μg/mL) were diluted in proportion to prepare six concentrations (X7–X12). Each concentration was tested twice, and values were averaged. According to the dilution ratio, a theoretical value for each sample was calculated (the linear range upper limit sample took the mean value of 2 repeated measurements as the theoretical value). The correlation coefficient r was obtained by regression analysis between the measured and theoretical values. Acceptance criteria were: kit detection in the range of 2.5 to 80 μg/mL; *r* ≥ 0.990.

#### 2.4.3 Measurement of FDP Detection Accuracy

We tested 53.0 μg/mL (high concentration samples, Z3) and 7.0 μg/mL (low concentration samples, Z4) three times each. The acceptance criterion was a relative deviation not exceeding ±10%.

#### 2.4.4 Determination of FDP Within‐Run Precision

The same batch of FDP kits was used to test high concentration samples (45.0 μg/mL) and low concentration samples (4.0 μg/mL). Each sample was tested 10 times in a row, and the coefficient of variation (CV) was calculated. The acceptance criterion was an intra‐batch CV ≤ 8.0%.

### 2.5 Determination of CYFRA21‐1 and FDP's Normal Reference Interval

SPSS 25.0 was used to analyze the normal distribution of the data grouped by different reference individuals. Normally distributed data would represent 95% of the data distribution range according to ±1.96 s; in the case of non‐normal distribution, a non‐parametric method was used, and the reference interval of FDP could be obtained by selecting the 95% quantile on one side.

### 2.6 Positive Judgment of CYFRA21‐1, FDP and Joint Detection

Receiver operating characteristic (ROC) curves were plotted for CYFRA21‐1 and FDP to assess their diagnostic performances in differentiating between bladder cancers and benign diseases or the healthy condition. Logistic regression analysis, with the presence of bladder cancer as the outcome and the levels of the biomarkers as the predictive variables, was performed to calculate the predicted probabilities of the biomarkers and their combination, which were used to estimate the respective ROC‐AUCs. The areas under the ROC curves (AUCs) of both tumor markers were compared.

### 2.7 Statistical Analysis

Data were analyzed with IBM SPSS Statistics 25.0 (IBM Corp., 2017). Normally and non‐normally distributed data were compared by the *t*‐test and the Mann–Whitney *U* test, respectively. The Kruskal–Wallis test was used to assess the correlation between TNM stage and urine FDP content in the bladder cancer group. The Mann–Whitney *U* test was used to determine the correlation between tumor grade and urine CYFRA21‐1 and FDP contents in the bladder cancer group. Two‐sided *p* < 0.05 was considered statistically significant. Finally, we performed logistic regression analysis to assess the associations of bladder cancer TNM stage, tumor infiltrating cells, and pathological grade with CYFRA21‐1 and FDP levels.

## 3 Results

### 3.1 Baseline Characteristics

The characteristics of the subjects included in this study are shown in Table 1. The proportion of males and patients older than 50 years was higher in the bladder cancer group compared with the total control group (75.71% vs. 56.68% and 91.43% vs. 41.06%, respectively). The median concentrations of urinary CYFRA21‐1 and FDP in the bladder cancer group were 14.030 ng/mL and 3.438 μg/mL, respectively, with significantly higher values in the bladder cancer group compared with the control group (*p* < 0.0001). Bladder cancer cases were divided into groups according to TNM stage and histological grade. The associations of CYFRA21‐1 and FDP levels with histopathological characteristics are shown in Table 2. The median detection levels of CYFRA21‐1 and FDP in the bladder cancer group trended to increase with disease course.

**TABLE 1:** Characteristics of the included subjects and CYFRA21‐1 and FDP levels.

Parameter	Bladder cancer (*n* = 70)	Control			*p* ^a^
		Healthy (*n* = 312)	Urinary calculi and infection (*n* = 85)	Total (*n* = 397)	
Sex					
No. of male, %	53, 75.71	172, 55.13	53, 62.35	225, 56.68	0.003
No. of female, %	17, 24.29	140, 44.87	32, 37.64	172, 43.32	
Age (years)^b^					
No. of ≥ 50, %	64, 91.43	123, 39.42	40, 47.06	163, 41.06	< 0.0001
No. of < 50, %	6, 8.57	189, 60.58	45, 52.94	234, 58.94	< 0.0001
CYFRA21‐1 (ng/mL)^b^	14.030 (6.376–57.238)	1.533 (0.549–2.372)	3.331 (1.111–8.792)	1.664 (0.587–2.816)	< 0.0001
FDP (μg/mL)^b^	3.438 (1.859–6.234)	1.357 (1.152–1.653)	1.770 (1.542–2.435)	1.443 (1.173–1.696)	< 0.0001

^a^ Bladder cancer group vs. total control group.

^b^ Results are expressed as median (1st to 3rd quartiles).

**TABLE 2:** Levels of corrected CYFRA21‐1 and FDP in various bladder cancer groups.

Classification (*n*)	CYFRA21‐1 (ng/mL)^a^	FDP (μg/mL)^a^
TNM		
TaN0M0 (14)	13.275 (6.616–36.900)	2.483 (1.424–3.440)
TisN0M0 (18)	7.792 (5.126–36.645)	2.251 (1.460–4.476)
T1N0M0 (16)	10.838 (5.066–52.295)	3.402 (1.938–5.681)
T2N0M0 (8)	17.590 (10.515–47.342)	4.973 (4.329–12.191)
T3N0M0 (11)	103.900 (49.260–162.800)	8.157 (3.058–19.380)
T4N2M0 (3)	4.984 (4.747–50.620)	37.730 (7.405–115.500)
*p* ^b^	0.022	
Tumor infiltrating cell type		
NMIBC (48)	9.988 (5.772–38.82)	2.841 (1.473–4.081)
MIBC (22)	49.940 (9.523–107.275)	7.157 (4.345–19.885)
*p* ^b^	0.011	< 0.0001
Pathological grade		
Low (32)	8.907 (4.773–27.790)	2.967 (1.496–4.173)
High (38)	36.420 (9.015–102.025)	4.604 (2.424–14.233)
*p* ^b^	0.006	0.006

^a^ Results are expressed as median (1st to 3rd quartiles).

^b^ *p* value for the difference among the subgroups.

### 3.2 CYFRA21‐1 Detection Performance

#### 3.2.1 CYFRA21‐1's LLOD Verification

A quantity fewer than three samples demonstrated concentrations beneath 0.1 ng/mL, and no specimens were detected with concentrations surpassing 3.3 ng/mL (Table 3).

**TABLE 3:** Verification of the detection limit for CYFRA21‐1 determination kit (ECLIA).

Sample number	Signal intensity	Detection value (ng/mL)
A1	2503	0.152
	2686	0.125
	2619	0.095
	2655	0.111
	2640	0.104
A2	2770	0.169
	2734	0.150
	2721	0.143
	2816	0.196
	2725	0.145
A3	2658	0.112
	2697	0.131
	2735	0.150
	2760	0.164
	2735	0.150
A4	2682	0.123
	2694	0.129
	2694	0.129
	2715	0.140
	2786	0.178
A5	2604	0.189
	2618	0.094
	2633	0.101
	2670	0.118
	2617	0.194
Conclusion	Qualified	

#### 3.2.2 CYFRA21‐1's Linear Range

**FIGURE 2:**
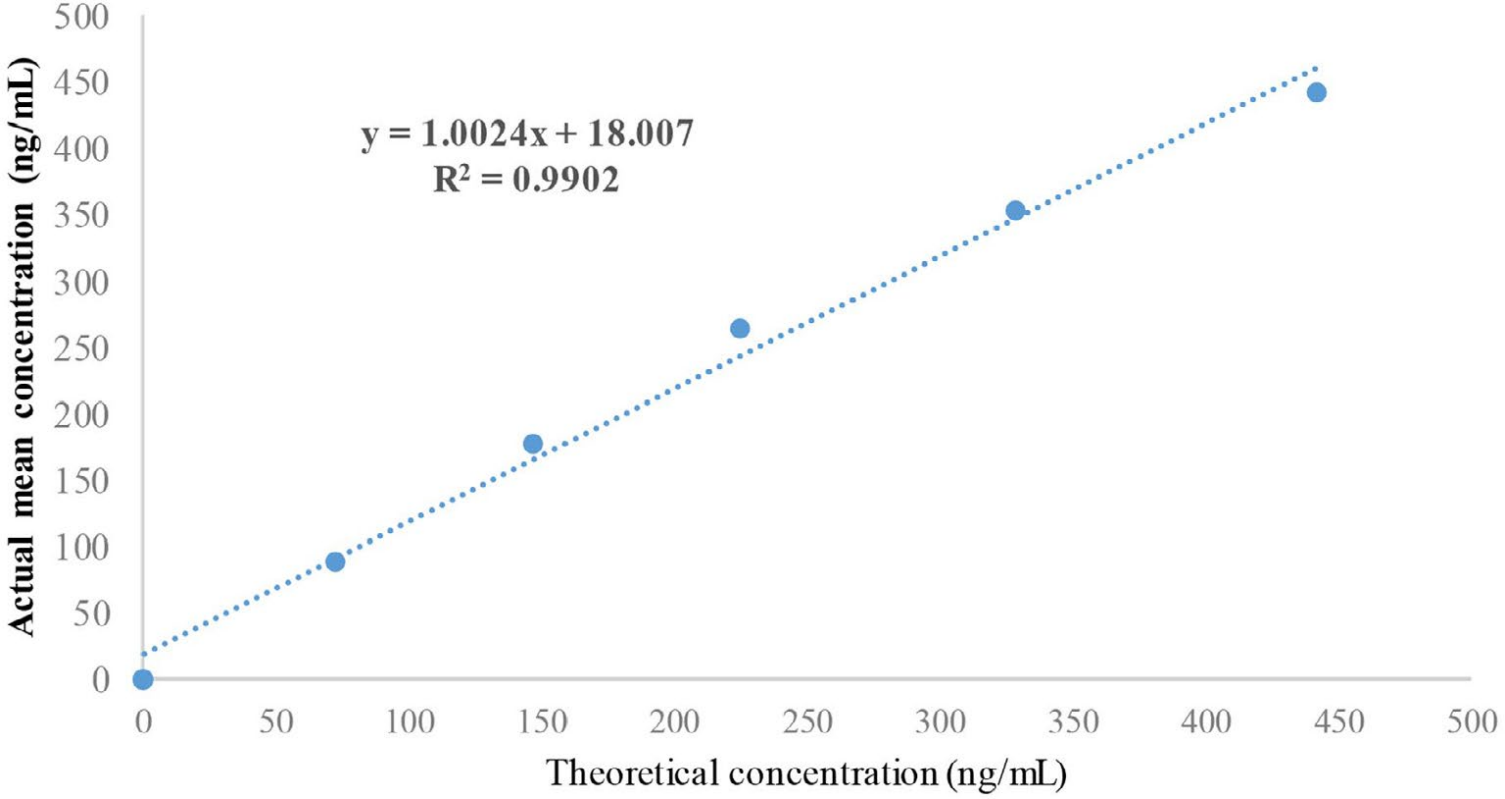
Linear range of the detection limit of the CYFRA21‐1 determination kit (ECLIA).

The regression equation for urinary CYFRA21‐1 detection with the CYFRA21‐1 kit in the linear range of 0.1–500 ng/mL was *y* = 1.0024*x* + 18.007, with a determination coefficient R^2^ of 0.990 and a correlation coefficient r of 0.9995 (*p* < 0.01), which met the validation requirements. The kit's accurate measurement range was 0.1–500 ng/mL (Figure 2).

#### 3.2.3 CYFRA21‐1's Accuracy

Table 4 shows that the relative deviations between the results measured for low‐value and high‐value samples and the target values were 0.526% and 0.000%, respectively, which met the verification requirements.

**TABLE 4:** Evaluation results of the accuracy of the CYFRA21‐1 detection.

Samples for testing accuracy	Detection value (ng/mL)	Actual mean value (ng/mL)	Target value (ng/mL)	Relative deviation (%)
Z1	36.965	37.265	37.070	0.526
	36.255			
	38.575			
Z2	3.124	3.047	3.047	0.000
	3.008			
	3.008			

#### 3.2.4 CYFRA21‐1 Detection's Within‐Run Precision

The coefficients of variation of the measured results for low‐value and high‐value samples were 1.287% and 1.469%, respectively, which were below 6.000% and met the validation requirements (Table 5).

**TABLE 5:** Evaluation results for intra‐batch precision of the CYFRA21‐1 determination kit (ECLIA).

Number	Minimum value (ng/mL)	Maximum value (ng/mL)
Test 1	3.150	37.343
Test 2	3.140	36.592
Test 3	3.083	36.861
Test 4	3.035	36.855
Test 5	3.060	36.357
Test 6	3.058	38.200
Test 7	3.133	37.282
Test 8	3.092	37.002
Test 9	3.109	37.380
Test 10	3.062	36.475
$\bar{X}$	3.092	37.035
*s*	0.040	0.544
CV	1.287%	1.469%
Acceptance standard	CV < 6%	

Abbreviations: $\bar{X}$, arithmetic mean; CV, coefficient of variation; *s*, standard deviation.

### 3.3 FDP Detection Performance Testing

#### 3.3.1 FDP's LLOD Verification

The detection results of 5 low value samples whose concentrations were close to the detection limit are shown in Table 6. The average values were all higher than 1.0 μg/mL and lower than 2.5 μg/mL; the detection limit was less than 2.5 μg/mL, indicating that these values met the verification requirements (Table 6).

**TABLE 6:** Verification results for the detection limit of the FDP determination kit (ECLIA).

Sample number	Signal intensity	Detection value (μg/mL)
A6	781	1.087
	790	1.098
	791	1.099
	791	1.099
	794	1.103
A7	794	1.103
	797	1.106
	797	1.106
	798	1.108
	776	1.108
A8	800	1.110
	801	1.111
	801	1.111
	801	1.111
	801	1.111
A9	802	1.112
	803	1.113
	804	1.115
	805	1.116
	807	1.119
A10	810	1.122
	813	1.125
	815	1.128
	819	1.133
	823	1.138
Conclusion	Qualified	

#### 3.3.2 FDP's Linear Range

**FIGURE 3:**
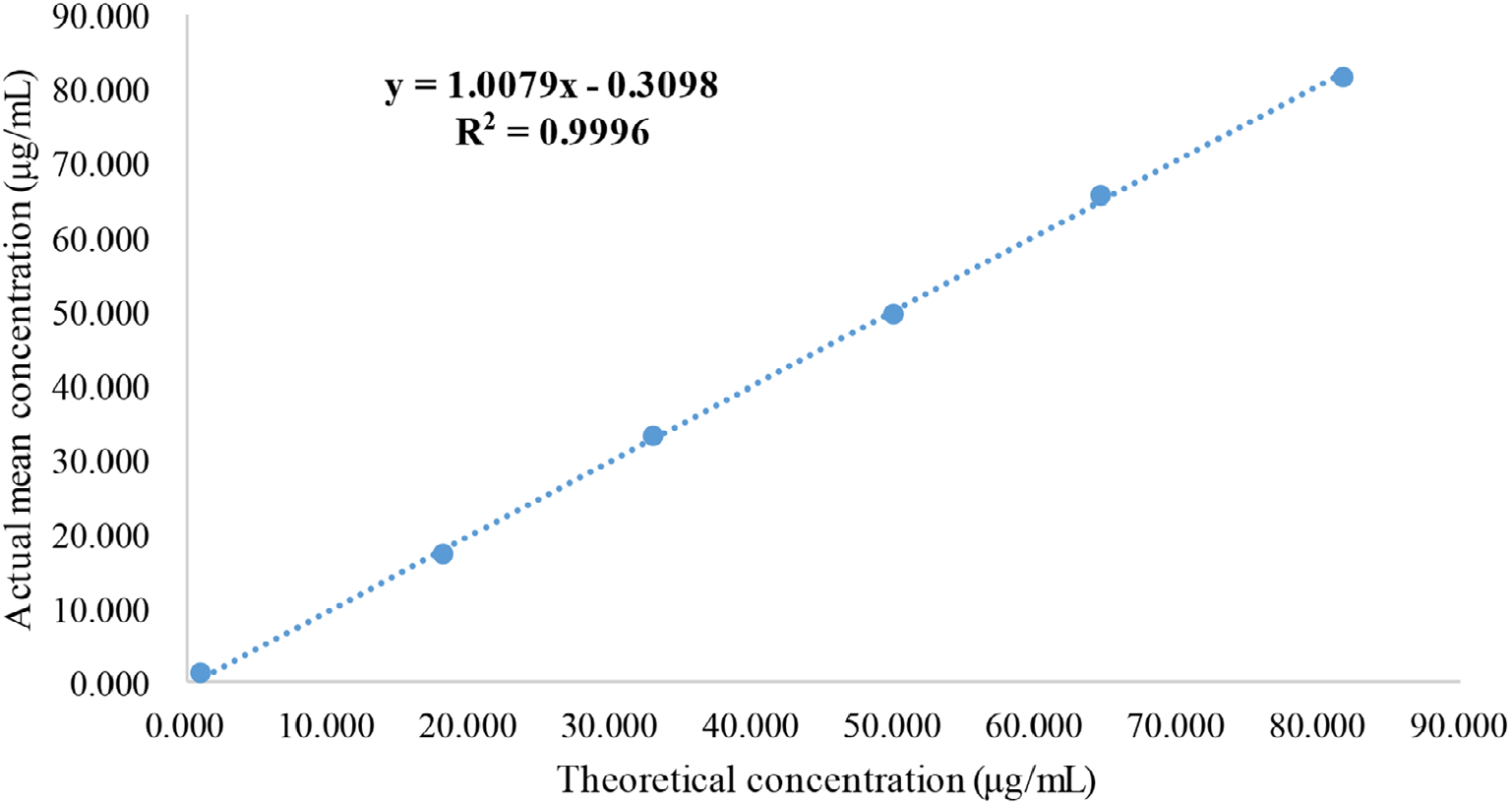
Linear range for the detection limit of the FDP determination kit (ECLIA).

The linear range test results for FDP are shown in Figure 3. The regression equation of urine FDP detected by the FDP kit in the linear range of 2.5–80 μg/mL was *y* = 1.0079*x* − 0.3098; *R* ^2^ was 0.9996 and *r* was 0.999 (*p* < 0.0001), which met the verification requirements (Figure 3).

#### 3.3.3 FDP Detection Accuracy

Table 7 shows that the relative deviations between the results measured for low‐value and high‐value samples and the target values were 0.21% and 0.273%, respectively, which met the verification requirements.

**TABLE 7:** Evaluation results for the accuracy of FDP detection.

Sample for testing accuracy	Detection value (μg/mL)	Actual mean value (μg/mL)	Target value (μg/mL)	Relative deviation (%)
Z3	51.890	51.607	51.500	0.002
	51.890			
	51.040			
Z4	7.141	7.180	7.200	−0.273
	7.171			
	7.229			

#### 3.3.4 FDP Detection's Within‐Run Precision

The coefficients of variation of 10 precision low‐value and high‐value samples were 0.498% and 4.135%, respectively, both of which were below 8%, which met the verification requirements (Table 8).

**TABLE 8:** Evaluation results for intra‐batch precision of the FDP determination kit (ECLIA).

Number	Low value (μg/mL)	High value (μg/mL)
Test 1	3.462	47.020
Test 2	3.423	40.640
Test 3	3.456	44.060
Test 4	3.438	45.200
Test 5	3.467	43.630
Test 6	3.438	45.340
Test 7	3.449	46.170
Test 8	3.476	44.030
Test 9	3.474	42.590
Test 10	3.447	43.620
$\bar{X}$	3.453	44.230
*s*	0.017	1.829
CV	0.498%	4.135%
Acceptance standard	CV < 8%	

Abbreviations: $\bar{X}$, arithmetic mean; CV, coefficient of variation; *s*, standard deviation.

### 3.4 CYFRA21‐1 and FDP's Normal Reference Interval

Urine CYFRA21‐1 data showed a non‐normal distribution and were evaluated by the 95% percentile method; the normal reference interval was less than 4.973 ng/mL. Urine FDP data also had a non‐normal distribution and were evaluated by the 95% percentile method; the normal reference interval was less than 1.739 μg/mL.

### 3.5 Values of FDP, CYFRA21‐1, and FDP + CYFRA21‐1 in Bladder Cancer Detection

**FIGURE 4:**
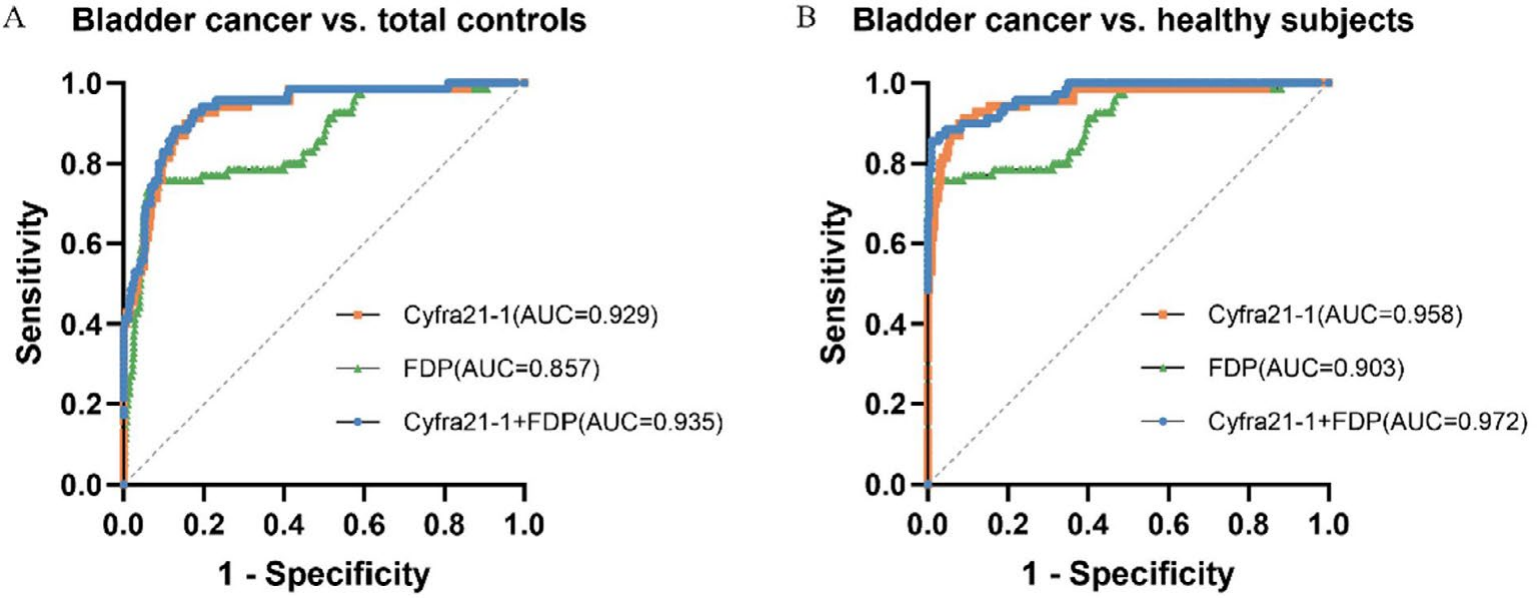
Diagnostic performances of FDP, CYFRA21‐1, and FDP + CYFRA21‐1 in predicting bladder cancer. (A) Receiver operating characteristic (ROC) curves for FDP, CYFRA21‐1, and FDP + CYFRA21‐1 for distinguishing the bladder cancer group (*n* = 70) from the total control group (*n* = 397). (B) ROC curves for the four markers in distinguishing bladder cancer cases (*n* = 70) from healthy subjects (*n* = 312).

The AUC values of CYFRA21‐1, FDP, and FDP + CYFRA21‐1 were 0.929, 0.857, and 0.935 in distinguishing bladder cancer (*n* = 70) and other benign diseases (*n* = 397), respectively (Figure 4A). Furthermore, the AUC values of CYFRA21‐1, FDP, and FDP + CYFRA21‐1 in identifying bladder cancer (*n* = 70) and healthy people (*n* = 312) were 0.958, 0.903, and 0.972, respectively (Figure 4B). The best cut‐off was determined when the sum of sensitivity and specificity was maximized. The established cut‐offs for differentiating the bladder cancer group from the total control group were 3.840 ng/mg and 2.173 μg/mg for CYFRA21‐1 and FDP, respectively. The resulting ROC‐AUCs, sensitivities, and specificities at the best cut‐offs are summarized in Table 9. When these cut‐off values were applied, the positive and negative predictive values in various groups were as follows: CYFRA21‐1, PPV = 49.23% and NPV = 96.78%; FDP, PPV = 65.00% and NPV = 95.34%; FDP + CYFRA21‐1, PPV = 55.37%, NPV = 97.75%.

**TABLE 9:** Sensitivities and specificities of CYFRA21‐1 and FDP tested independently and in combination.

Paremater	Bladder cancer (*n* = 70) vs. total controls (*n* = 397)				Bladder cancer (*n* = 70) vs. healthy subjects (*n* = 312)				*α* (%)	*β* (%)	PPV (%)	NPV (%)
	ROC‐AUC^a^	*p* ^b^	Sensitivity (%)^a^	Specificity (%)^a^	ROC‐AUC^a^	*p* ^b^	Sensitivity (%)^a^	Specificity (%)^a^				
CYFRA21‐1	0.929 (0.897 to 0.962)	< 0.0001	91.43 (82.53 to 96.01)	83.38 (79.40 to 86.71)	0.958 (0.929 to 0.987)	< 0.0001	91.43 (82.53 to 96.01)	91.35 (87.70 to 93.98)	16.62	15.71	49.23	96.78
FDP	0.857 (0.805 to 0.910)	< 0.0001	74.29 (62.97 to 83.07)	92.95 (90.00 to 95.08)	0.903 (0.858 to 0.949)	< 0.0001	75.71 (64.50 to 84.25)	99.36 (97.69 to 99.89)	7.05	25.71	65.00	95.34
CYFRA21‐1 + FDP	0.935 (0.905 to 0.966)	< 0.0001	88.57 (79.04 to 94.09)	87.41 (83.78 to 90.31)	0.972 (0.953 to 0.991)	< 0.0001	85.71 (75.66 to 92.05)	99.04 (97.12 to 99.74)	12.59	11.43	55.37	97.75

Abbreviations: *α*, mis‐diagnostic rate; *β*, false negative diagnostic rate; NPV, negative predictive value; PPV, positive predictive value; ROC‐AUC, area under the receiver operating characteristic curve.

^a^ Value (95% confidence interval).

^b^ *p* value for the difference between negative and positive samples for CYFRA 21‐1 and/or FDP.

### 3.6 Statistical Analysis

**FIGURE 5:**
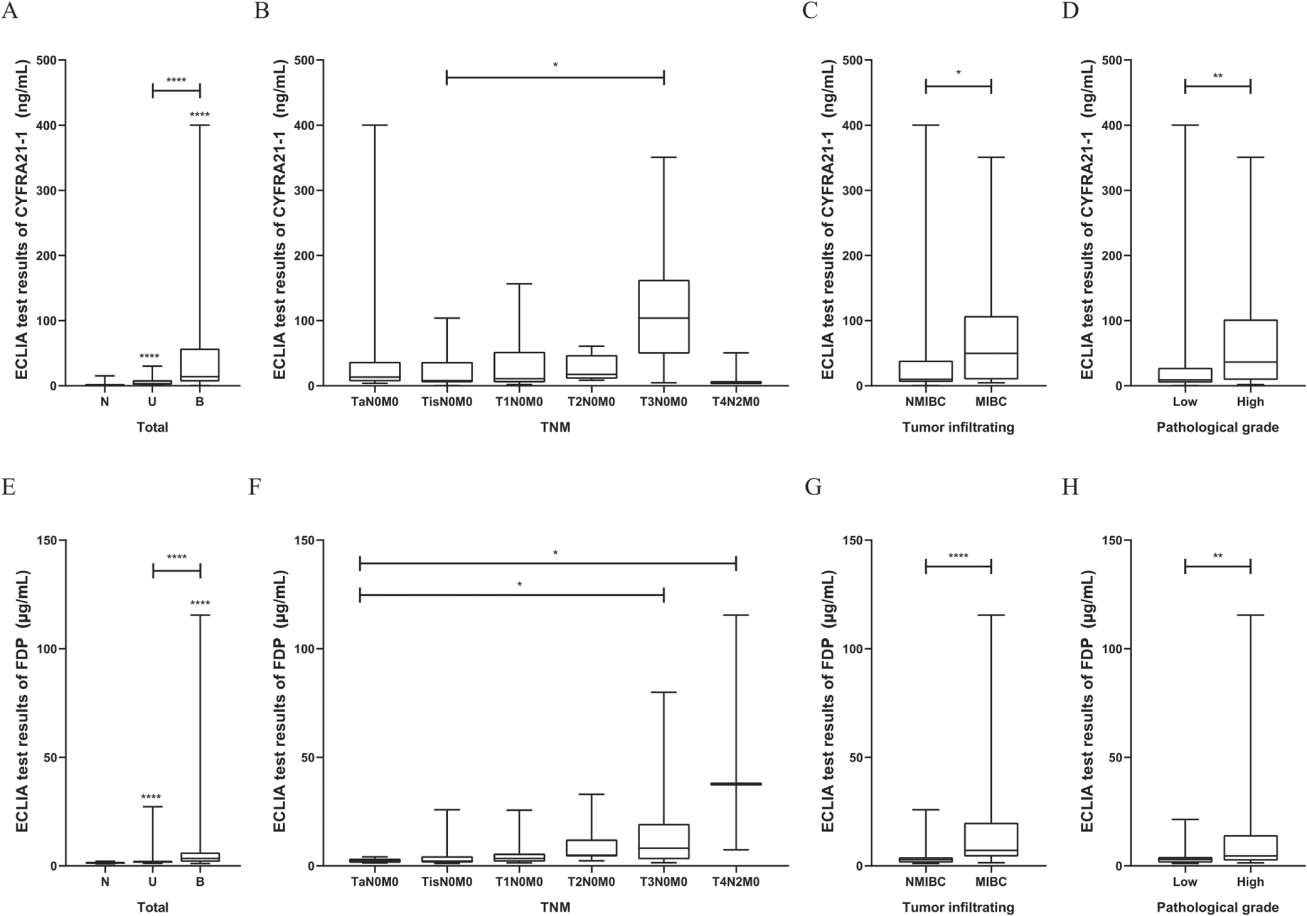
Correlation analyses of CYFRA21‐1 and FDP. B, bladder cancer group; N, healthy control group; U, urinary calculi and infection group. ****p < 0.0001, ***p < 0.001, **p < 0.01, *p < 0.05. (A) Urinary CYFRA21‐1 levels in the healthy control, urological inflammatory disease, and bladder cancer groups. (B) Urinary CYFRA21‐1 levels in different bladder cancer groups based on TNM stage. (C) Urinary CYFRA21‐1 levels in bladder cancer groups based on tumor infiltrating cells. (D) Urinary CYFRA21‐1 levels in bladder cancer groups based on pathological grade. (E) Urinary FDP levels in the healthy control, urinary calculi and infection, and bladder cancer groups. (F) Urinary FDP levels in bladder cancer groups based on TNM stage. (G) Urinary FDP levels in bladder cancer groups based on tumor infiltrating cells. (H) Urinary FDP levels in bladder cancer groups based on pathological grade.

The results of CYFRA21‐1 and FDP were non‐normally distributed, and the differences between the groups for both indexes were analyzed by the Mann–Whitney *U* test, respectively, and the differences were statistically significant (*p* < 0.0001) (Figure 5A,E). The associations of TNM stage with CYFRA21‐1 and FDP levels in the detected urine in the bladder cancer group were analyzed by the Kruskal–Wallis test. The difference between TisN0M0 (*n* = 18) and T3N0M0 (*n* = 11) in CYFRA21‐1 results was significant (*p* = 0.011, *Z* = −3.379) (Figure 5B). The difference between TaN0M0 (*n* = 14) and T3N0M0 (*n* = 11) in FDP results was significant (*p* = 0.020, *Z* = −3.208) (Figure 5F). The difference between TaN0M0 (*n* = 14) and T4N0M0 (*n* = 3) in FDP results was significant (*p* = 0.020, *Z* = −3.209) (Figure 5F). The associations of pathological grade and infiltration degree with urine CYFRA21‐1 and FDP levels in the bladder cancer group were analyzed by the Mann–Whitney *U* test. For CYFRA21‐1 detection, the difference in the degree of tumor invasion was statistically significant (*p* = 0.011, *Z* = 2.549) (Figure 5C). There were significant differences among different grades of bladder cancer (*p* = 0.006, *Z* = −2.735) (Figure 5D). For FDP detection, there were statistically significant differences in the degree of tumor invasion (*p* < 0.0001, *Z* = −4.263; Figure 5G) and among different grades of bladder cancer (*p* = 0.006, *Z* = −2.737; Figure 5H). This suggested that urinary CYFRA21‐1 and FDP detected by ECLIA could effectively distinguish bladder cancer samples from non‐bladder cancer samples, as well as muscular invasive bladder cancer samples and non‐muscular invasive bladder cancer samples.

In a regression analysis of CYFRA21‐1 and FDP detection levels as predictors, the OR values of the correlation between CYFRA21‐1 and TNM stage, degree of invasion, and pathological grade of bladder cancer were 1.005, 1.006, and 1.007, respectively, while the OR values of the correlation between FDP and TNM stage, degree of invasion, and pathological grade of bladder cancer were 1.137, 1.140, and 1.152, respectively. (Table 10).

**TABLE 10:** Results of Multivariate logistic regression analysis.

Covariate	CYFRA21‐1				FDP			
	OR	SE	95% CI	*p*	OR	SE	95% CI	*p*
TNM	1.005	0.004	0.997 to 1.013	0.152	1.137	0.049	1.048 to 1.275	0.009
Tumor infiltrating cells	1.006	0.004	0.999 to 1.015	0.101	1.140	0.049	1.051 to 1.277	0.007
Pathological grade	1.007	0.004	0.999 to 1.018	0.144	1.152	0.067	1.039 to 1.357	0.034

Abbreviations: CI, confidence interval; OR, odds ratio; SE, standard error.

## 4 Discussion

The classic clinical methods for the early diagnosis of bladder cancer are cystoscopy and urine exfoliative cytology, which are affected by objective conditions such as testing instruments and the experience of attending doctors [6, 22]. Identifying tumor markers in urine may be a noninvasive and convenient method of diagnosing bladder cancer early on. Among them, BTA, NMP22, FDP, and other urine markers have been approved by the United States Food and Drug Administration (FDA) for clinical use. CYFRA21‐1 was initially designed to detect lung cancer [23], in our previous study, it showed a good potential in distinguishing bladder cancer cases from the control group [24]. In the past decade, new urine detection methods have shown their own disadvantages; for example, fluorescence in situ hybridization (FISH) has the disadvantage of complex and low‐throughput operation [25]. TERT promoter mutations in the urine of bladder cancer patients could be detected by droplet digital PCR in recent years [26]. Although this method has the advantages of low preparation difficulty and high detection flux, its linear range is small, and part of the dead volume is easy to cause detection result deviations when separating liquid and metastasis [22, 26]. Meanwhile, as for methylation's use in detecting bladder cancer in urine, it is easily affected by the interethnic living environment and habits [22, 27]. A considerable number of reports have evaluated the diagnostic effectiveness of the latex agglutination method and ELISA for CYFRA21‐1 and FDP [16, 28]. Recently, however, few reports have discussed the effectiveness of ECLIA in the detection of CYFRA21‐1 and FDP in urine. In this study, urinary CYFRA21‐1 and FDP detection by ECLIA had good differentiation potential in bladder cancer. The application of ECLIA kits and the ECLIA platform also greatly reduces the detection time, with a detection time of only 9 min per sample, realizing the automation and standardization of detection. In addition, most of the previous studies only evaluated individual markers, marking it difficult to compare these markers for diagnostic effectiveness and to apply them in clinic. In this case, we simultaneously evaluated the effectiveness of CYFRA21‐1 and FDP in the same subjects.

Kit performance testing confirmed that the CYFRA21‐1 and FDP kits have good precision, wide linear range, excellent trueness, intra‐assay precision, and high specificity, and fully satisfied the requirements for clinical testing. However, we found that there might be a certain timeliness of the FDP antigen in urine, so the results of the FDP immediate test were more accurate.

Urine markers of bladder cancer often show low specificity in some benign diseases, including inflammation, infection, stones, and so on [27, 29, 30]. At present, a single urine marker cannot fill this gap; therefore, by combining urine markers, bladder tumor markers would be more specific and clinically effective. The median levels of both markers were higher in the bladder cancer group than in the control group, consistent with another study [16]. It was previously reported that urinary calculi and urinary tract infections cause abnormal results for tumor markers, even in those without bladder cancer [31]. So, the total control group consisted of urolithiasis cases, urinary infection cases, and healthy patients. However, both CYFRA21‐1 and FDP levels were slightly higher in urolithiasis and infection patients and bladder cancer patients than in healthy controls (*p* < 0.0001). CYFRA21‐1 detection may yield false negative results in detecting samples with positive low values (3.840–4.973 ng/mL); FDP may be more effective in detecting samples with negative high values (1.739–2.173 μg/mL), yielding false positive results.

As shown above, the ROC‐AUCs of CYFRA21‐1, FDP, and CYFRA21‐1 + FDP for bladder cancer detection were 0.929, 0.857, and 0.935, which were higher than reported by Jeong et al. (0.90, 0.77 and 0.88, respectively) [16]. Recently, a study explored proteomic and glycomic biomarkers with high sensitivity and specificity for bladder cancer [32]. The authors set different sensitivity/specificity criteria (sensitivity and specificity should be ≥ 90% vs. sensitivity and specificity of at least one marker in the combination should be ≥ 90%) according to the type of marker (single marker vs. combined marker). Notably, the ROC‐AUC of CYFRA21‐1 and FDP jointly detected by the ECLIA method was higher than that of each single indicator. Its AUC (0.935) was significantly higher than the reference threshold (0.90) in the literature. This suggests that the combination of CYFRA21‐1 and FDP may yield a better diagnostic performance. The balance of sensitivity and specificity of the diagnostic model was more in line with the actual clinical needs. In addition, the synergistic effect of noninvasive detection methods and markers further highlights the application potential of this joint inspection strategy. In the process, AUC may be affected by differences in patient populations and control groups in different studies; the validity of these markers should be interpreted with caution.

In this study, FDP was significantly correlated with TaN0M0 and T3N0M0 stages, tumor grade, and tumor invasion, while CYFRA21‐1 was significantly correlated with TisN0M0 and T3N0M0 stages, tumor grade, and tumor invasion. But the OR is 1, illustrating that CYFRA21‐1 is not a risk factor for disease progression. While FDP is a risk factor for TNM stage, degree of invasion, and pathological grade of bladder cancer. Each unit increase in FDP was associated with a 13.7% increased risk of TNM, a 14.0% increased risk of tumor invasion, and a 15.2% increased risk of tumor grade progression, which is different from what has been previously reported in the literature and may be a result of the effect of differences in patient populations and control groups in different studies. In this study, most patients were diagnosed with non‐muscle invasive bladder cancer.

The current study design had some limitations, and most bladder cancer subjects had superficial bladder cancer, making it difficult to verify the diagnostic efficacies of CYFRA21‐1 and FDP in advanced bladder cancer. Urine markers should be chosen according to the clinical conditions to take advantage of their full potential. Given the short stability period of fibrin degradation products (FDP) in urine samples, subsequent experiments will focus on optimizing the stabilizers in FDP detection kits. This modification is anticipated to enhance the stability of measurement outcomes and improve clinical reliability. Additionally, we plan to increase the sample size in each experimental group to mitigate potential bias in statistical analysis caused by coexisting conditions such as bladder calculi and urinary tract inflammation. Furthermore, our ongoing research will integrate advanced imaging technologies with deep learning‐based recognition algorithms, aiming to develop a computer‐aided diagnostic system that could significantly improve the diagnostic accuracy for bladder cancer.

## 5 Conclusions

Bladder cancer, as a common urological malignant disease, does not have an ideal marker in terms of early screening with disease course monitoring. The continued necessity of developing noninvasive diagnostic means for bladder cancer has guided the production of various urinary tumor markers. In this study, our systematic evaluation evaluated the laboratory diagnostic capability of the combined detection of CYFRA21‐1 and FDP in bladder cancer by electrochemiluminescence assay, and the analysis indicated its good discriminatory potential in the diagnosis of bladder cancer. This suggests that the combined detection of urinary CYFRA21‐1 and FDP by ECLIA is expected to be a more effective means to identify bladder carcinogenesis.

## Author Contributions

**Zhuoran Li:** conceptualization, methodology, validation, investigation, formal analysis, data curation, writing – original draft, writing – review and editing, visualization, supervision, project administration. **Lu Xia:** conceptualization, methodology, investigation, writing – review and editing, visualization, supervision, project administration. **Yamin Li:** conceptualization, methodology, investigation, resources, writing – review and editing, supervision, project administration. **Tao Huang:** investigation, writing – review and editing, visualization. **Ning Yang:** investigation, writing – review and editing, visualization. **Shannai Li:** investigation, writing – review and editing, visualization. **Yinyin Luo:** investigation, writing – review and editing, visualization. **Yaoyao Yang:** investigation, writing – review and editing, visualization. **Jian Zhang:** investigation, writing – review and editing, visualization. **Song Wu:** conceptualization, methodology, investigation, resources, writing – review and editing, visualization, supervision, project administration, funding acquisition. **Qifang Lei:** conceptualization, methodology, investigation, resources, writing – review and editing, visualization, supervision, project administration, funding acquisition.

## Ethics Statement

The study was approved by the Shenzhen Luohu People's Hospital's Ethics Committee (2019‐LHQRMYY‐LL‐053).

## Consent

Patients gave written informed consent to participate.

## Conflicts of Interest

The authors declare no conflicts of interest.

## Data Availability

All data generated or analyzed during this study are included in this published article.
